# Hydrogen Sulfide in Paraventricular Nucleus Enhances Sympathetic Activity and Cardiac Sympathetic Afferent Reflex in Chronic Heart Failure Rats

**DOI:** 10.1371/journal.pone.0050102

**Published:** 2012-11-15

**Authors:** Xian-Bing Gan, Tong-Yan Liu, Xiao-Qing Xiong, Wei-Wei Chen, Ye-Bo Zhou, Guo-Qing Zhu

**Affiliations:** 1 Key Laboratory of Cardiovascular Disease and Molecular Intervention, Department of Physiology, Nanjing Medical University, Nanjing, China; 2 Department of Physiology, Anhui University of Traditional Chinese Medicine, Hefei, China; University of Buenos Aires, Cardiovascular Pathophysiology Institute, Argentina

## Abstract

**Background:**

Intracerebroventricular infusion of NaHS, a hydrogen sulfide (H_2_S) donor, increased mean arterial pressure (MAP). This study was designed to determine the roles of H_2_S in the paraventricular nucleus (PVN) in modulating sympathetic activity and cardiac sympathetic afferent reflex (CSAR) in chronic heart failure (CHF).

**Methodology/Principal Findings:**

CHF was induced by left descending coronary artery ligation in rats. Renal sympathetic nerve activity (RSNA) and MAP were recorded under anesthesia. CSAR was evaluated by the RSNA and MAP responses to epicardial application of capsaicin. PVN microinjection of low doses of a H_2_S donor, GYY4137 (0.01 and 0.1 nmol), had no significant effects on RSNA, MAP and CSAR. High doses of GYY4137 (1, 2 and 4 nmol) increased baseline RSNA, MAP and heart rate (HR), and enhanced CSAR. The effects were greater in CHF rats than sham-operated rats. A cystathionine-β-synthase (CBS) inhibitor, hydroxylamine (HA) in PVN had no significant effect on the RSNA, MAP and CSAR. CBS activity and H_2_S level in the PVN were decreased in CHF rats. No significant difference in CBS level in PVN was found between sham-operated rats and CHF rats. Stimulation of cardiac sympathetic afferents with capsaicin decreased CBS activity and H_2_S level in the PVN in both sham-operated rats and CHF rats.

**Conclusions:**

Exogenous H_2_S in PVN increases RSNA, MAP and HR, and enhances CSAR. The effects are greater in CHF rats than those in sham-operated rats. Endogenous H_2_S in PVN is not responsible for the sympathetic activation and enhanced CSAR in CHF rats.

## Introduction

Chronic heart failure (CHF) is characterized by sympathetic activation [1]. The excessive sympathetic activity not only deteriorates CHF, but also is prognostic of death and complications [2], [3]. Cardiac sympathetic afferent reflex (CSAR) is a sympatho-excitatory reflex, which can be induced by stimulation of cardiac sympathetic afferents with exogenous chemicals or endogenous chemicals released from myocardium during myocardial ischemia [4]. The enhanced CSAR is involved in the sympathetic over-activation in CHF [5]–[7].

Hydrogen sulfide (H_2_S) is known as a physiologically important gaseous transmitter that is endogenously produced to influence biological functions such as anti-oxidation, anti-inflammation, neuromodulation and cardiovascular actions in mammalian [8]. H_2_S is largely produced from L-cysteine (Cys) and homocysteine (Hcy) by the actions of cystathionine-β-synthase (CBS) and cystathionine-γ-lyase (CSE). CBS is primarily found in the central nervous system, whereas CSE is mainly expressed in the peripheral tissues [8]. H_2_S has been reported to have positive properties such as cardioprotective effects against cardiac ischemia-reperfusion injury [9], and antihypertensive effects via peripheral vasodilatory actions [10]. Infusion of sodium hydrosulfide (NaHS), a H_2_S donor, into lateral cerebral ventricle of rats increases mean arterial pressure (MAP) [11]. Bilateral microinjection of NaHS into rostral ventrolateral medulla (RVLM) decreases MAP and renal sympathetic nerve activity (RSNA) in rats, while hydroxylamine (HA), a CBS inhibitor, increases MAP and RSNA [12]. These findings indicate that opposite cardiovascular effects of H_2_S can be observed in different brain regions.

Paraventricular nucleus (PVN) of hypothalamus is an integrative site in regulating sympathetic and cardiovascular activity [13], [14], and is a component of central neurocircuitry of the CSAR [15], [16]. Previous studies have shown that PVN is involved in excessive sympathetic activation and enhanced CSAR in CHF [17]–[20]. CBS expression has been found in the PVN [21]. An interesting question is whether H_2_S in the PVN contributes to sympathetic activation and enhanced CSAR in CHF. The present study was designed to determine the roles of H_2_S in the PVN in regulating sympathetic nerve activity and CSAR in normal rats and CHF rats.

## Materials and Methods

Experiments were carried out on male Sprague-Dawley rats weighing between 300 and 400 g. The procedures were approved by the Experimental Animal Care and Use Committee of Nanjing Medical University (No. 20110451) and complied with the Guide for the Care and Use of Laboratory Animals (NIH publication no. 85–23, revised 1996). The rats were kept in a temperature-controlled room with a 12 h–12 h light-dark cycle, with standard chow and tap water ad libitum.

### CHF Model

CHF was induced by coronary artery ligation as previously described [7], [22]. Briefly, rats were anesthetized with sodium pentobarbital (50 mg kg^−1^, i.p.) and were instrumented using sterile techniques. The rats were randomly subjected to the ligation of the left anterior descending coronary artery or sham operation. The sham-operated (Sham) rats were treated the same as the coronary ligation rats except their coronary arteries were not ligated. The criterion for CHF was that left ventricle end-diastolic pressure (LVEDP) was higher than 12 mm Hg. At the end of experiment, the atria and right ventricular free wall was removed. The left ventricle was opened with an incision along the septum from base to apex so that the tissue could be pressed flat. The circumferences of the left ventricle (LV) and the region of infarcted tissue were outlined on a photograph taken by a digital camera and measured with SigmaScan program (SPSS Science). Infarct size was calculated and expressed as a percentage of the LV surface area [23], [24].

### General Procedures of Acute Experiment

Acute experiment was carried out 7 weeks after coronary ligation or sham surgery. Each rat was anaesthetized with intraperitoneal injection of urethane (800 mg kg^−1^) and α-chloralose (40 mg kg^−1^). Supplemental doses of anesthetic agents were administered to maintain an adequate depth of anesthesia during experiments. A midline incision was made to expose the trachea and carotid artery. The trachea was connected with a rodent ventilator (683; Harvard Apparatus Inc., USA) for mechanical ventilation. A catheter connected to a pressure transducer was placed into the right carotid artery for blood pressure recordings.

### RSNA Recording

Left renal sympathetic nerve was isolated through a retroperitoneal incision. The nerve was cut distally to eliminate its afferent activity. A pair of silver electrodes was placed on the nerve and immersed in warm mineral oil. The signal was amplified (× 1000) with an AC/DC differential amplifier (3000; A–M Systems Inc., Sequim, WA, USA) and filtered with a band-pass between 60 and 3000 Hz. The amplified and filtered signal was integrated at a time constant of 100 ms. At the end of each experiment, the background noise was determined after section of the central end of the nerve and was subtracted from the integrated values of the RSNA [25]. The raw and integrated RSNA, arterial blood pressure (ABP), MAP and heart rate (HR) were simultaneously recorded with a PowerLab data acquisition system (8SP; ADInstruments, Sydney, Australia).

### Evaluation of CSAR

A left lateral thoracotomy was performed and the pericardium was removed. The CSAR was induced by epicardial application of a piece of filter paper (3 mm×3 mm) containing capsaicin (1.0 nmol in 2.0 µl) to the non-infarct area of the left ventricle. The CSAR was evaluated by the RSNA and MAP responses to epicardial application of capsaicin [7], [26].

### PVN Microinjection

Rat was fixed in a stereotaxic frame (Stoelting, Chicago, IL, USA). The coordinates for the PVN is 1.8 mm caudal from bregma, 0.4 mm lateral to the midline, and 7.9 mm ventral to the dorsal surface according to the stereotaxic atlas of Paxinos & Watson [27]. The microinjection volume was 50 nl in each side of the PVN. At the end of the experiment, the same volume of 2% Evans Blue was injected into each microinjection site for histological identification. The rat was excluded from data analysis if the distance between the centre point of microinjection and the boundary of the PVN was less than 0.15 mm [28].

### Measurement of H_2_S Level, CBS Level and CBS Activity

Samples were rapidly collected and frozen in liquid nitrogen as previously reported [29]. Protein concentrations in the supernatant were measured using a protein assay kit (BCA, Pierce). H_2_S levels in the PVN were measured using a rat H_2_S ELISA kit (NovaTeinBio, Cambridge, USA) according to the manufacturer’s instructions. Briefly, a 96-well microplate was coated with an antibody specific for H_2_S. Standard and samples were transferred to assay plate, incubated at 37°C and then washed. Subsequently, horseradish peroxidase (HRP)-conjugated reagent was added, incubated and then washed. Chromogenic solution were added and kept in the dark at 37°C. Then, stop solution was added to stop the reaction. The optical density (OD) was measured at 450 nm using an ELISA plate reader (ELX-800, BioTeK, Winooski, USA).

CBS levels in the PVN were measured using a rat CBS ELISA kit (Antibodies-Online Inc., Atlanta, USA) according to the manufacturer’s instructions. Antibody specific for CBS was pre-coated onto a microplate. Standards and samples were pipetted into the wells and any CBS present was bound to the immobilized antibody. After removing any unbound substances, a biotin-conjugated antibody specific for CBS was added to the wells. After washing, avidin conjugated HRP was added to the wells. Following a wash to remove any unbound avidin-enzyme reagent, a substrate solution is added to the wells and color develops in proportion to the amount of CBS bound in the initial step. The color development is stopped and the intensity of the color is measured at 450 nm.

For CBS activity assays, the tissues were homogenized in buffer containing 30 mM potassium phosphate, 1 mM beta-mercaptoethanol and 1∶50 protease inhibitor cocktail. The homogenate was centrifuged at 20,000×g for 10 min. The supernatant was assayed as reported previously [30], [31]. One unit of CBS activity catalyzes the formation of 1 µmol of cystathionine in 1 h at 37°C.

### Chemicals

Morpholin-4-ium 4 methoxyphenyl(morpholino) phosphinodithioate (GYY4137) was obtained from Cayman Chemical (Ann Arbor, Michigan, USA). Hydroxylamine (HA), capsaicin and DMSO were purchased from Sigma Chemical (St. Louis, MO, USA). GYY4137 and HA were dissolved in normal saline containing 1% DMSO just before microinjection. Capsaicin was dissolved in normal saline.

### Experimental Design

#### Experiment 1

Normal rats were randomly divided into three groups (n = 6 for each group) to determine the time effects of 1% DMSO, 2 nmol of GYY4137 (a H_2_S donor) and 3 nmol of HA (a CBS inhibitor) in the PVN on RSNA, MAP and HR.

#### Experiment 2

Random microinjection of different doses of GYY4137 (0, 0.01, 0.1, 1, 2 or 4 nmol) into the PVN were carried out to determine the dose effect of GYY4137 on baseline RSNA and MAP, and CSAR in 6 Sham rats and 6 CHF rats. The interval between microinjections was at least 40 min for complete recovery. The baseline changes were determined by averaging 1 min of the parameters at the fourth minute after each microinjection. The CSAR induced by epicardial application of capsaicin was tested 4 min after each microinjection. The maximal RSNA and MAP responses to capsaicin were determined by averaging 30 sec of the parameters. Similar procedures were used to determine the dose effect of HA (0, 0.3 or 3 nmol) in the PVN on baseline RSNA and MAP, and CSAR in 6 Sham rats and 6 CHF rats.

#### Experiment 3

Effects of epicardial application of capsaicin to stimulate cardiac afferents on CBS activity, CBS level and H_2_S level in the PVN were determined. Either Sham rats or CHF rats were randomly divided into two groups, which were subjected to successive epicardial application of saline or capsaicin three times lasting 1 min for each application (n = 6 for each group). Then, the samples were quickly collected for the measurements using ELISA method.

#### Experiment 4

H_2_S levels were determined after microinjection of GYY4137 into the PVN to evaluate the H_2_S release from GYY4137 in vivo. Normal rats were randomly divided into seven groups (n = 3 for each group). Six groups of rats were subjected to bilateral PVN microinjection of 1% DMSO or different doses of GYY4137 (0.01, 0.1, 1, 2 or 4 nmol). Another group of rats was used as blank control (Ctrl). The samples were quickly collected 4 min after microinjections. H_2_S levels of left and right side of the PVN were separately measured using ELISA method.

### Statistics

Comparisons between two observations in the same animal were assessed by Student’s paired *t* test. One-way or two-way ANOVA followed by the Bonferroni test for post hoc analysis was used when multiple comparisons were made. The values were expressed as the mean±SE. *P*<0.05 was considered statistically significant.

## Results

### Anatomical and Hemodynamic Data

Mean infarct area in CHF rats was 30.1% of the LV, and no obvious infarct was found in Sham rats. The heart weight and heart-to-body weight ratio were increased in CHF rats. Systolic arterial pressure, LV peak systolic pressure (LVSP) and the maximum of the first differentiation of LV pressure (+dP/dt_max_) decreased, but the LVEDP increased in CHF rats (Table 1).

**Table 1:** Anatomic and hemodynamic data in Sham and CHF rats.

	Sham	CHF
n	24	24
Body weight (g)	359.0±5.4	357.1±5.6
Heart weight (g)	1.3±0.04	1.6±0.04*
Heart weight/body weight (g/kg)	3.6±0.1	4.6±0.1*
Infarct size (% LV area)	0	30.1±2.0*
Systolic pressure (mm Hg)	125.5±2.9	112.2±2.2*
Diastolic pressure (mm Hg)	81.5±2.6	81.8±2.9
Mean arterial pressure (MAP, mm Hg)	97.2±2.7	91.9±2.4
Heart rate (HR, beats/min)	352.0±7.8	369.0±15.6
LV systolic pressure (LVSP, mm Hg)	136.3±4.9	115.4±2.7*
LV end-diastolic pressure (LVEDP, mm Hg)	0.9±1.0	15.8±0.6*
+LV dP/dt_max_ (mm Hg/sec)	3558±144	1918±102*

LV, left ventricle;+LV dP/dtmax, maximum of the first differentiation of LV pressure. Values are mean ± SE.

* P<0.05 compared with Sham rats.

### Time Effects of GYY4137 and HA

**Figure 1:**
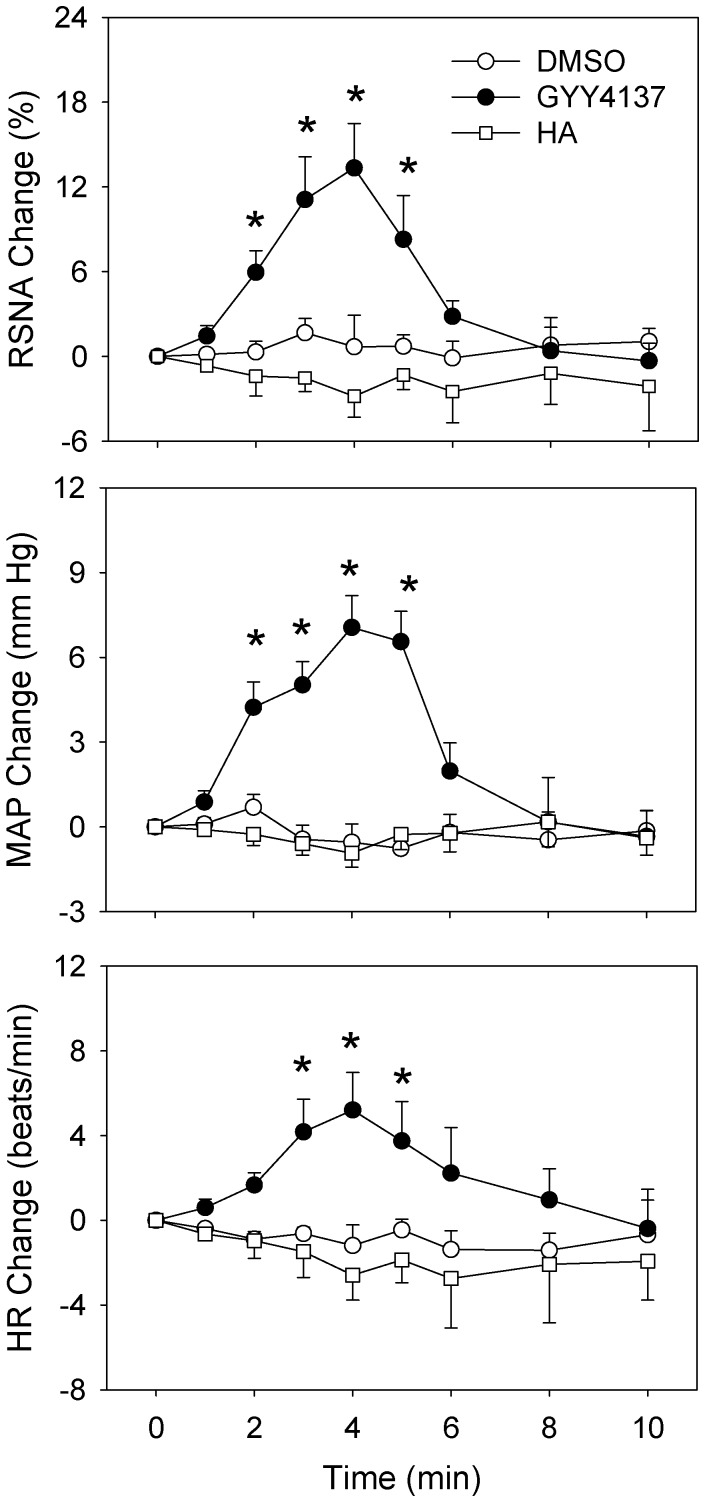
Baseline RSNA, MAP and HR changes caused by PVN microinjection of 1% DMSO, GYY4137 (2 nmol) or HA (3 nmol) in normal rats. n = 6 for each group. Values are mean±SE. * P<0.05 compared with DMSO.

Microinjection of GYY4137, a H_2_S donor, into the PVN caused immediate increases in the RSNA, MAP and HR, peaking at about 4 min in normal rats. The effects of GYY4137 lasted about 8 min (Fig. 1). Microinjection of HA (a CBS inhibitor) into the PVN had no significant effects on the RSNA, MAP and HR (Fig. 1).

### Effects of Different Dose of GYY4137 and HA on Baseline RSNA, MAP and HR

**Figure 2:**
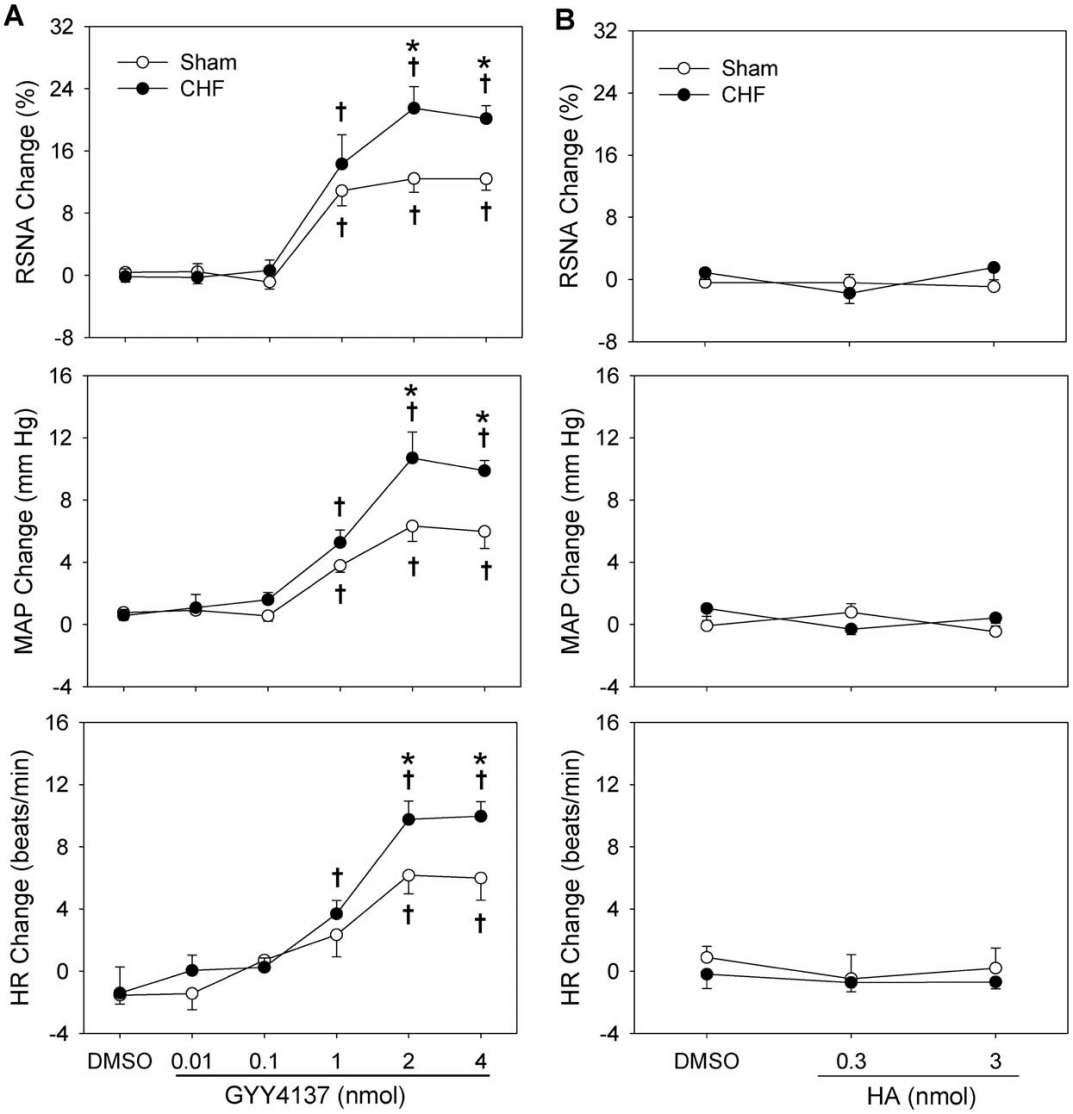
Effects of PVN microinjection of 1% DMSO, different doses of GYY4137 (A), or HA B) on baseline RSNA, MAP and HR in Sham and CHF rats. n = 6 for each group. Values are mean±SE. *P<0.05 compared with Sham rats; †P<0.05 compared with DMSO.

Microinjection of 0.01 or 0.1 nmol of GYY4137 into the PVN had no significant effects on baseline RSNA, MAP and HR, while 1, 2 or 4 nmol of GYY4137 increased baseline RSNA, MAP and HR significantly in both Sham and CHF rats. The effects of GYY4137 were much greater in CHF rats than that in Sham rats. However, 4 nmol of GYY4137 failed to cause greater effects than 2 nmol of GYY4137. No significant effect of HA was found in both Sham and CHF rats (Fig. 2).

### Effects of Different Dose of GYY4137 and HA on CSAR

**Figure 3:**
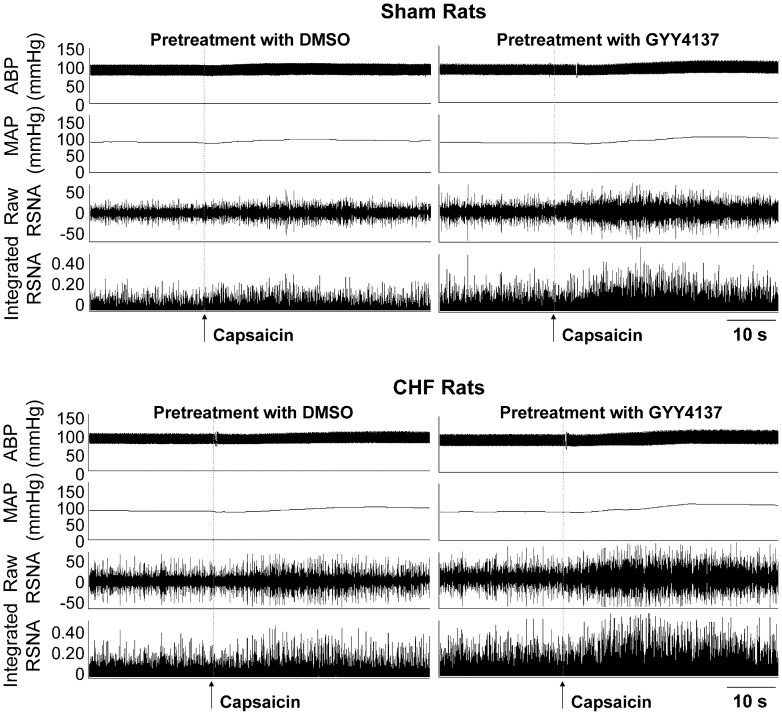
Representative tracings showing the effect of PVN pretreatment with 1% DMSO or GYY4137 on the CSAR in Sham and CHF rats. The CSAR was evaluated by the RSNA responses to epicardial application of capsaicin.

**Figure 4:**
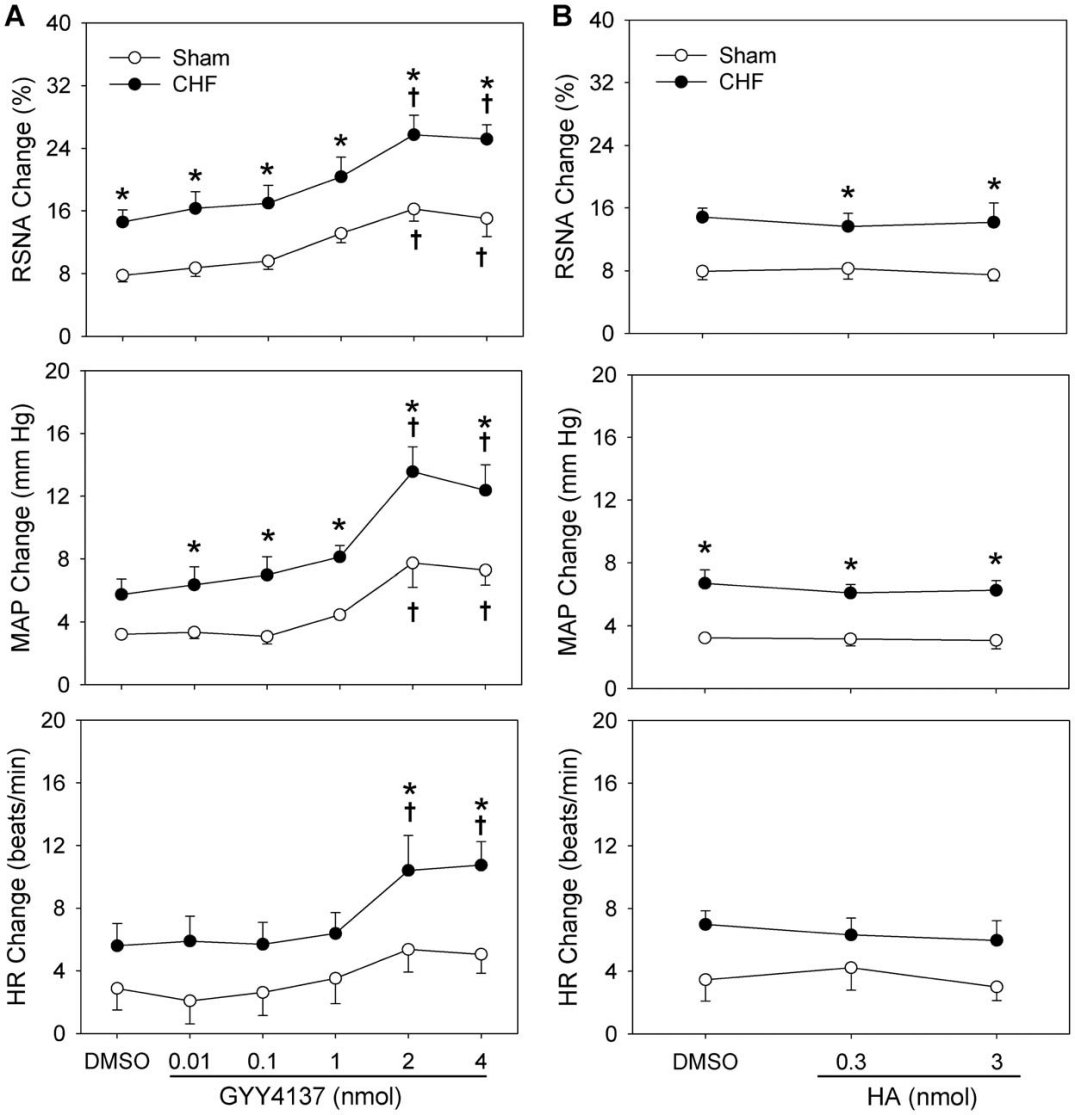
Effects of PVN microinjection of 1% DMSO, different doses of GYY4137 (A), or HA (B) on capsaicin-induced CSAR in Sham and CHF rats. n = 6 for each group. Values are mean±SE. *P<0.05 compared with Sham rats; †P<0.05 compared with DMSO.

Representative traces showed that PVN microinjection of 4 nmol of GYY4137 enhanced the capsaicin-induced CSAR in both Sham and CHF rats (Fig. 3). Microinjection of 0.01, 0.1 or 1 nmol of GYY4137 into the PVN had no significant effects on CSAR, while 2 or 4 nmol of GYY4137 significantly enhanced the CSAR. The effect of GYY4137 on the CSAR was much greater in CHF rats than that in Sham rats. However, 4 nmol of GYY4137 failed to cause greater effects than 2 nmol of GYY4137. No significant effect of HA on the CSAR was found in both Sham and CHF rats (Fig. 4).

### CBS Activity, CBS Level and H_2_S Level in PVN

**Figure 5:**
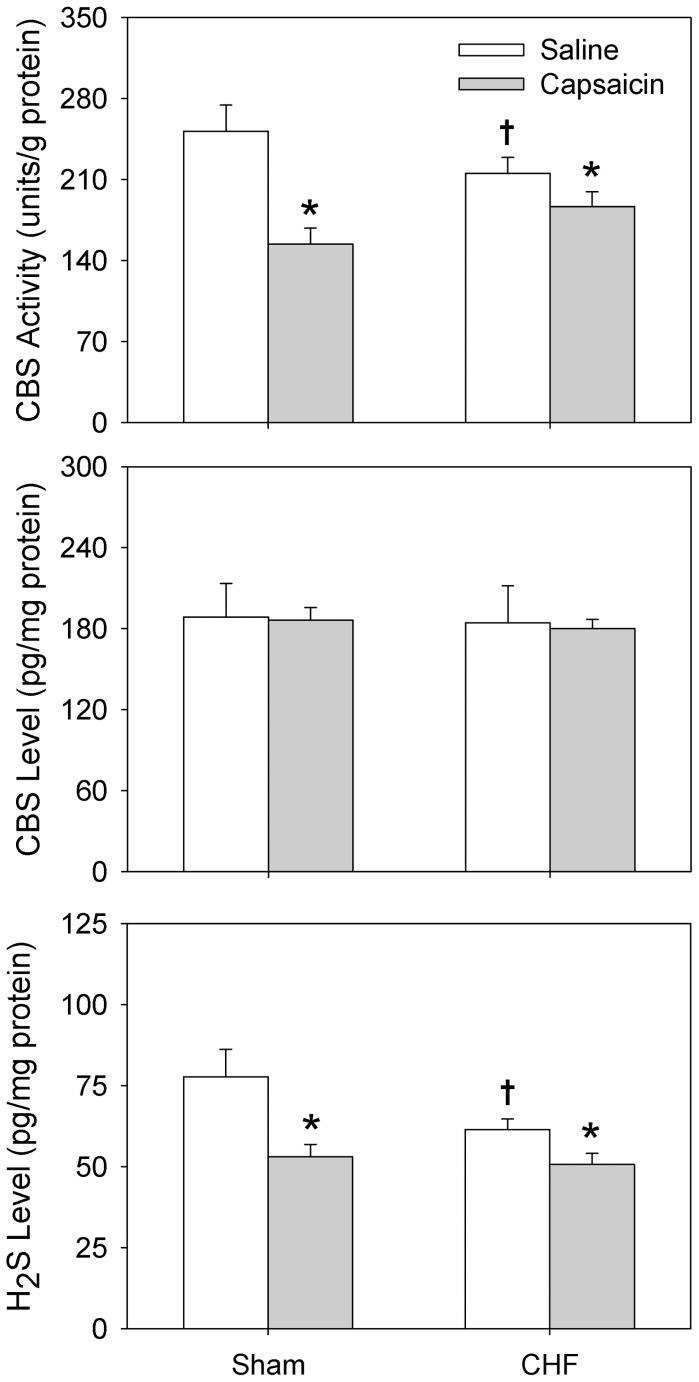
Effects of epicardial application of saline or capsaicin on the CBS activity, CBS level and H_2_S level in PVN in Sham and CHF rats. n = 6 for each group. Values are mean±SE. *P<0.05 compared with Saline; †P<0.05 compared with Sham rats.

CBS activity and H_2_S level in the PVN were decreased in CHF rats treated with saline compared with Sham rats treated with saline. Epicardial application of capsaicin significantly decreased the CBS activity and H_2_S level in the PVN in both Sham rats and CHF rats. However, there was no significant difference in the CBS level of the PVN between Sham rats and CHF rats (Fig. 5).

### H_2_S Release from GYY4137

**Figure 6:**
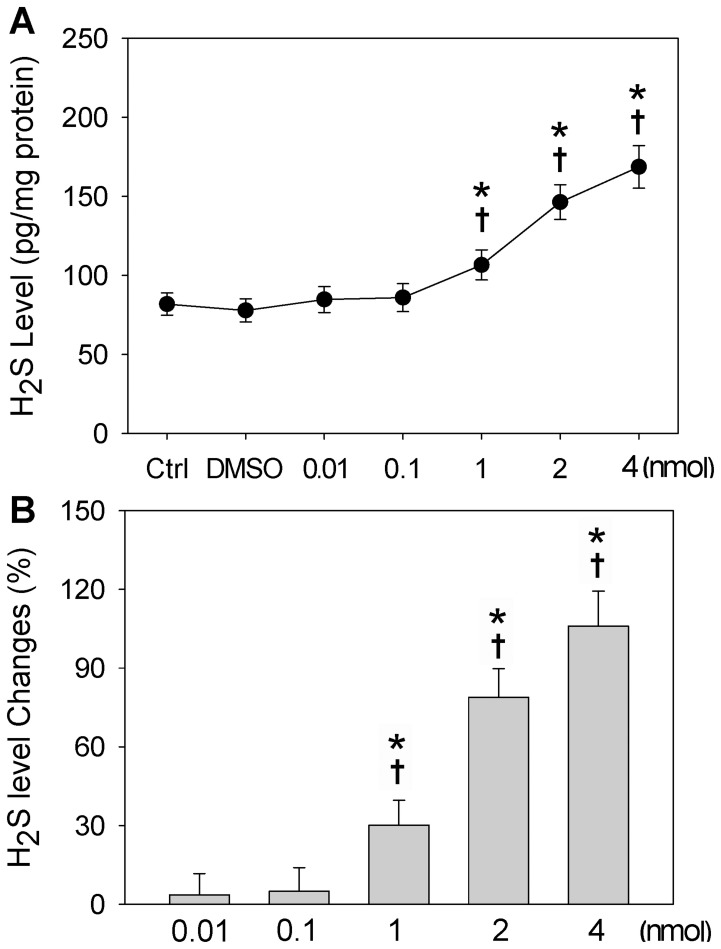
H_2_S levels in PVN in normal rats. n = 6 for each group. A, H_2_S levels in blank control rats (Ctrl) and in rats treated with PVN microinjection of 1% DMSO or GYY4137 (0.01, 0.1, 1, 2 or 4 nmol). Values are mean±SE. *P<0.05 compared with Ctrl; †P<0.05 compared with DMSO. B, Percentage change of H_2_S levels in the PVN caused by PVN microinjection of GYY4137 (0.01, 0.1, 1, 2 or 4 nmol). *P<0.05 compared with 0.01 nmol of GYY4137; †P<0.05 compared with 0.1 nmol of GYY4137.

Compared with Ctrl or DMSO, 0.01 or 0.1 nmol of GYY4137 in the PVN failed to increase the H_2_S level in the PVN, while 1, 2 or 4 nmol of GYY4137 in the PVN caused a dose-related increase in the H_2_S level of the PVN in normal rats (Fig. 6).

## Discussion

The primary findings are that a high dose of GYY4137, a H_2_S donor, in the PVN enhances the CSAR and increases baseline RSNA, MAP and HR, and that the effects of GYY4137 were greater in CHF rats than Sham rats. Inhibition of the CBS with HA in the PVN had no significant effects in both Sham and CHF rats. CBS activity and H_2_S level in the PVN decreased in CHF rats. Epicardial application of capsaicin to stimulate cardiac afferents decreased the CBS activity and H_2_S level in the PVN in both Sham and CHF rats. These results indicate that exogenous H_2_S in the PVN caused greater increases in sympathetic outflow and enhancement in the CSAR in CHF rats than Sham rats. Endogenous H_2_S in the PVN is not responsible for the excessive sympathetic activity and CSAR in CHF rats.

Coronary ligation induced CHF model simulates the most common cause of CHF in humans and permits precise timing of the inciting event and of the changes in neurohumoral systems and left ventricular function as CHF progresses [32]. The reduced arterial systolic pressure (ASP), LVSP and+LV dP/dt_max_ as well as increased LVEDP in CHF rats showed an impaired contractile function in CHF rats. The decrease in ASP in CHF rats is relevant to the reduced LVSP and ejection fraction due to impaired contractile function. However, the difference between the LVSP and ASP is greater in Sham rats (10.8 mm Hg) than that in CHF rats (3.2 mm Hg). A possible explantation is that some compensatory mechanisms may play a role in preventing hypotension in CHF state, which is supported by the data that arterial diastolic pressure and MAP were not significantly decreased in CHF rats. An important mechanism is that excessive sympathetic activation and high plasma norepinephrine level in CHF state [1] increase total peripheral resistance and maintain blood pressure.

H_2_S is cell-permeant and soluble in both water and organic solvents, which serves as a gaseous mediator in peripheral organs and brain [8]–[11]. NaHS is used as a H_2_S donor in many previous studies. However, NaHS is a short-lived and unstable donor and does not mimic the slow and continuous process of H_2_S generation in vivo [33], [34]. Furthermore, NaHS in aqueous solutions can be rapidly oxidized by oxygen [35]. It has been reported that microinjection of NaHS into the RVLM decreased ABP, HR, and RSNA [12]. Almost at the same time, another study has shown that microinjection of NaHS into the RVLM and PVN has no significant effects on MAP, HR and lumbar sympathetic nerve activity (LSNA) and is concluded that H_2_S in the RVLM and PVN has no effect on cardiovascular regulation [21]. The inconsistent results are probably caused by the instability of NaHS. A few years ago, we have tried many times to test the effects of NaHS in the PVN and RVLM on MAP and RSNA. We found that it is difficult to get stable effects of NaHS on MAP and RSNA in vivo (unpublished data). Modifications that are made between the time that a solution is prepared and the time that the biological effect is measured can dramatically affect the results [35]. Due to several serious drawbacks in using NaHS as a H_2_S donor, some investigators were focused on looking for better H_2_S donor used for H_2_S research. In 2008, a novel, water-soluble H_2_S-releasing molecule GYY4137 has been identified as a slow-releasing H_2_S compound, which effectively avoids the shortcoming of NaHS [33]. GYY4137 becomes a useful tool in the study of the many and varied biological effects of H_2_S recently [36]–[41].

In the present study, the novel H_2_S donor GYY4137 was used to investigate the effects of H_2_S in the PVN. Microinjection of a higher dose of GYY4137 increased baseline RSNA, MAP and HR, and enhanced the CSAR in both Sham and CHF rats. The effects were greater in CHF rats than those in Sham rats. These results suggest that exogenous H_2_S in the PVN regulates sympathetic activity and CSAR in both Sham and CHF rats. Sensitivity of PVN to H_2_S is increased in CHF rats. Previous studies have indicated that the CSAR increases sympathetic activity and blood pressure in either normal rats or CHF rats [4], [5]. We speculate that the CSAR-enhancing effect of GYY4137 at least partially contributes the increases in RSNA, MAP and HR in both Sham and CHF rats.

CBS level primarily depends on the CBS gene expression, while CBS activity is highly regulated. CBS contains a heme cofactor that functions as a redox sensor and utilizes S-adenosylmethionine (SAM) as an allosteric activator. CBS activity is a crucial factor for the H_2_S production [42], [43]. It is out of our expectation that the CBS activity and H_2_S level in the PVN were reduced in CHF rats, and no significant difference in the CBS level in PVN was found between Sham and CHF rats. Inhibition of CBS with HA failed to cause any significant effects on the baseline RSNA, MAP and HR, or the CSAR in both Sham and CHF rats. The results suggest that endogenous H_2_S in the PVN is not involved in the tonic control of sympathetic outflow and CSAR in normal rats, and is not responsible for the excessive sympathetic activity and enhanced CSAR in CHF rats. The differences in CBS activity and H_2_S level, not in CBS level, between Sham and CHF rats, indicated that the change of H_2_S level in CHF rats was mainly related to the altered CBS activity rather than CBS gene expression. It is interesting that stimulating cardiac afferents with capsaicin to induce the CSAR caused reduction of CBS activity and H_2_S level in the PVN in both Sham and CHF rats. The result indicates that the CSAR is not mediated by endogenous H_2_S in the PVN. On the other hand, it seems as if the results had a conflict with the finding that exogenous H_2_S in the PVN enhances the CSAR. A possible explanation is that exogenous H_2_S inhibits the production or the effects of nitric oxide (NO) in the PVN, which is supported by the following findings. H_2_S inhibits the vasorelaxant effect of NO presumably by forming a molecule (possibly a nitrosothiol), which exhibits little or no vasorelaxant activity [44]. NO synthase (NOS) inhibitors in the PVN increased sympathetic activity and blood pressure while NO donor caused a depressor effect [45]–[47]. The CSAR in CHF is enhanced by intracerebroventricular injection of NOS inhibitors and inhibited by NO donor [48]. However, more evidences are needed to support the interaction of H_2_S and NO in the PVN in regulation of the CSAR and sympathetic activity. Previous study showed that infusion of HA into the RVLM (1.5 nmol) significantly increased RSNA, ABP and HR [12]. In the present study, both doses of HA (0.3 and 3.0 nmol) failed to show any significant effects. In our preliminary study, we found that very high dose of HA (10.0 nmol) had no obvious effects (n = 2, data was not shown). We consider that insignificant effects of HA were not due to the insufficient dose of HA.

It is noted that the effects of GYY4137 were not in exact dose-dependent manner in the entire dose range (0.01–4 nmol). Low dose of GYY4137 (0.01 or 0.1 nmol) had no significant effects, while very high dose of GYY4137 (4 nmol) failed to cause greater effects than high dose of GYY4137 (2 nmol). In order to understand the extraordinary dose-effect relationship of GYY4137, we measured the H_2_S level in the PVN after the PVN microinjection of different doses of GYY4137. It was found that a low dose of GYY4137 (0.01 or 0.1 nmol) failed to increase H_2_S level, which may be the reason that the low doses of GYY4137 had no significant effects. It is possible that the small amount of H_2_S released from a low dose of GYY4137 is rapidly diffused away or converted to inactivated forms. H_2_S levels were significantly increased in a dose-dependent manner when the doses of GYY4137 exceeded 1 nmol, which were consistent with the effects of GYY4137 within the range. A very high dose of GYY4137 (4 nmol) caused higher H_2_S level in the PVN, but failed to augment its biological effects further. A possible explantation is that 2 nmol of GYY4137 had reached its maximal effects on RSNA, MAP, HR and CSAR. In addition, biological effects of GYY4137 only lasted about 8 min in vivo in the present study. These results also suggest that the doses of GYY4137 and time windows should be cautiously selected in experimental design, and should be taken into serious consideration in analyzing the results from GYY4137.

It has been previously shown that the firings of capsaicin-sensitive neurons in the PVN are greater in rats with baroreceptor denervation and vagotomy than that in intact rats after epicardial application of capsaicin, while electrical stimulation of the vagal afferents inhibits the response to capsaicin. The CSAR and the CSAR responses to angiotensin II in the PVN are greater in intact CHF rats than the rats with baroreceptor denervation and vagotomy [7]. The present study was carried out in rats with intact innervation, which is more accord with actual situation in CHF. There is a possibility that the baroreceptor and vagal afferent activity could interfere with the obtained results in intact rats.

In conclusion, exogenous H_2_S in the PVN increased sympathetic outflow, ABP and HR, and enhance the CSAR in both Sham rats and CHF rats. The effects were greater in CHF rats than Sham rats. Endogenous H_2_S in the PVN is not responsible for the excessive sympathetic activity and CSAR in CHF rats.
